# Incorporating domain knowledge in chemical and biomedical named entity recognition with word representations

**DOI:** 10.1186/1758-2946-7-S1-S9

**Published:** 2015-01-19

**Authors:** Tsendsuren Munkhdalai, Meijing Li, Khuyagbaatar Batsuren, Hyeon Ah Park, Nak Hyeon Choi, Keun Ho Ryu

**Affiliations:** 1 Database/Bioinformatics Laboratory, School of Electrical & Computer Engineering, Chungbuk National University, Cheongju, South Korea

**Keywords:** Feature Representation Learning, Semi-Supervised Learning, Named Entity Recognition, Conditional Random Fields

## Abstract

**Background:**

Chemical and biomedical Named Entity Recognition (NER) is an essential prerequisite task before effective text mining can begin for biochemical-text data. Exploiting unlabeled text data to leverage system performance has been an active and challenging research topic in text mining due to the recent growth in the amount of biomedical literature.

We present a semi-supervised learning method that efficiently exploits unlabeled data in order to incorporate domain knowledge into a named entity recognition model and to leverage system performance. The proposed method includes Natural Language Processing (NLP) tasks for text preprocessing, learning word representation features from a large amount of text data for feature extraction, and conditional random fields for token classification. Other than the free text in the domain, the proposed method does not rely on any lexicon nor any dictionary in order to keep the system applicable to other NER tasks in bio-text data.

**Results:**

We extended BANNER, a biomedical NER system, with the proposed method. This yields an integrated system that can be applied to chemical and drug NER or biomedical NER. We call our branch of the BANNER system BANNER-CHEMDNER, which is scalable over millions of documents, processing about 530 documents per minute, is configurable via XML, and can be plugged into other systems by using the BANNER Unstructured Information Management Architecture (UIMA) interface.

BANNER-CHEMDNER achieved an 85.68% and an 86.47% F-measure on the testing sets of CHEMDNER Chemical Entity Mention (CEM) and Chemical Document Indexing (CDI) subtasks, respectively, and achieved an 87.04% F-measure on the official testing set of the BioCreative II gene mention task, showing remarkable performance in both chemical and biomedical NER. BANNER-CHEMDNER system is available at: https://bitbucket.org/tsendeemts/banner-chemdner.

## Background

As biomedical literature on servers grows exponentially in the form of semi-structured documents, biomedical text mining has been intensively investigated to find information in a more accurate and efficient manner. One essential task in developing such an information extraction system is the Named Entity Recognition (NER) process, which basically defines the boundaries between typical words and biomedical terminology in a particular text, and assigns the terminology to specific categories based on domain knowledge.

NER performance in the newswire domain is indistinguishable from human performance, because it has an accuracy that is above 90%. However, performance has not been the same in the biomedical and chemical domain. It has been hampered by problems such as the number of new terms being created on a regular basis, the lack of standardization of technical terms between authors, and often by the fact that technical terms, such as gene names, often occur with other terminologies [1].

Proposed solutions include rule-based, dictionary-based, and Machine Learning (ML) approaches. In the dictionary-based approach, a prepared terminology list is matched through a given text to retrieve chunks containing the location of the terminology words [2,3]. However, medical and chemical text can contain new terminology that has yet to be included in the dictionary.

The rule-based approach defines particular rules by observing the general features of the entities in a text [4]. In order to identify any named entity in text data, a rule-generation process has to process a huge amount of text to collect accurate rules. In addition, the rules are usually collected by domain experts, requiring a lot of effort.

Since the machine learning approach was adopted, significant progress in biomedical and chemical NER has been achieved with methods like the Markov Model [5], the Support Vector Machine (SVM) [6-8] the Maximum Entropy Markov Model [9,10], and Conditional Random Fields (CRF) [2,11-13]. However, most of the studies rely on supervised machine learning, and thus, system performance is limited by the training set that is usually built by a domain expert. Studies have shown that the word, the word *n*-gram and the character *n*-gram, and the traditional orthographic features are the base for NER, but are poor at representing domain background.

In order to incorporate domain knowledge into an ML model, Semi-Supervised Learning (SSL) techniques have been applied to NER. SSL is an ML approach that typically uses a large amount of unlabeled and a small amount of labeled data to build a more accurate classification model than would be built using only labeled data. SSL has received significant attention for two reasons. First, preparing a large amount of data for training requires a lot of time and effort. Second, since SSL exploits unlabeled data, the accuracy of classifiers is generally improved. There have been two different directions in SSL methods: 1) semi-supervised model induction approaches, which are the traditional methods and which incorporate domain knowledge from unlabeled data into the classification model during the training phase [14,15], and 2) supervised model induction with unsupervised, possibly semi-supervised, feature learning. The approaches in the second research direction induce better feature representation by learning from a large unlabeled corpus. Recently, the studies that apply the word representation features induced on the large text corpus have reported improvement over baseline systems in many Natural Language Processing tasks [16-18].

Several BioCreative shared tasks have been organized in order to evaluate researcher advancements in chemical and biomedical text mining [19,20]. BioCreative IV CHEMDNER Track consists of two subtasks: the Chemical Document Indexing subtask, where participants are asked to provide a ranked list of chemical entities found in each of the PubMed documents, and the Chemical Entity Mention recognition subtask, where they are asked to submit the start and end indices corresponding to all the chemical entities mentioned in a particular document [21]. This study extends our previous work participated in CHEMDNER task in the following ways. First, the unlabeled data used to build unsupervised models is now enriched with a large collection of PMC articles. Second, we induce word vector class models from word vectors as word representations. Third, we explore several variations of the unsupervised models built with a larger text collection than the one used before. Finally, we take a step towards a unified NER system in biomedical, chemical and medical domain by training and evaluating a biomedical NER model. These changes lead an improvement outperforming our official entries for CHEMDNER CEM and CDI subtasks by a 0.93% and a 0.73% F-measure, respectively.

In order to incorporate domain knowledge into the machine learning model to leverage overall system performance, we propose a semi-supervised learning method that efficiently exploits unlabeled data. The proposed method includes NLP tasks for text preprocessing and learning word representation features from a large amount of raw text data, in addition to the word, the word *n*-gram, the character *n*-gram, and the traditional orthographic information (baseline features) for feature extraction, and applies CRF for token labeling. Our method does not rely on any lexicon, nor any dictionary other than the free text in the domain, in order to keep the system applicable to the other NER tasks in bio-text data, even though the usage of such resources is reported to considerably boost system performance.

During the development, we extended the BANNER system [22] with the proposed method, since that system is used in many biomedical text mining systems [23-26], showing state-of-the-art performance in biomedical Named Entity Recognition. BANNER extracts the most fundamental features for NER such as orthographic, letter *n-gram *and word prefix features, builds on top of a CRF model, and includes two types of postprocessing rule, namely parenthesis matching and abbreviations resolving. Our extension yields an integrated system that can be applied to chemical and drug NER or biomedical NER. We call our branch of the BANNER system BANNER-CHEMDNER, which is scalable and configurable, and can easily be plugged into other systems. BANNER-CHEMDNER shows an 85.68% and an 86.47% F-measure on the testing sets of CHEMDNER CEM and CDI subtasks, respectively, and an 87.04% F-measure on the official testing set of the BioCreative II gene mention task.

## Methods

**Figure 1:**
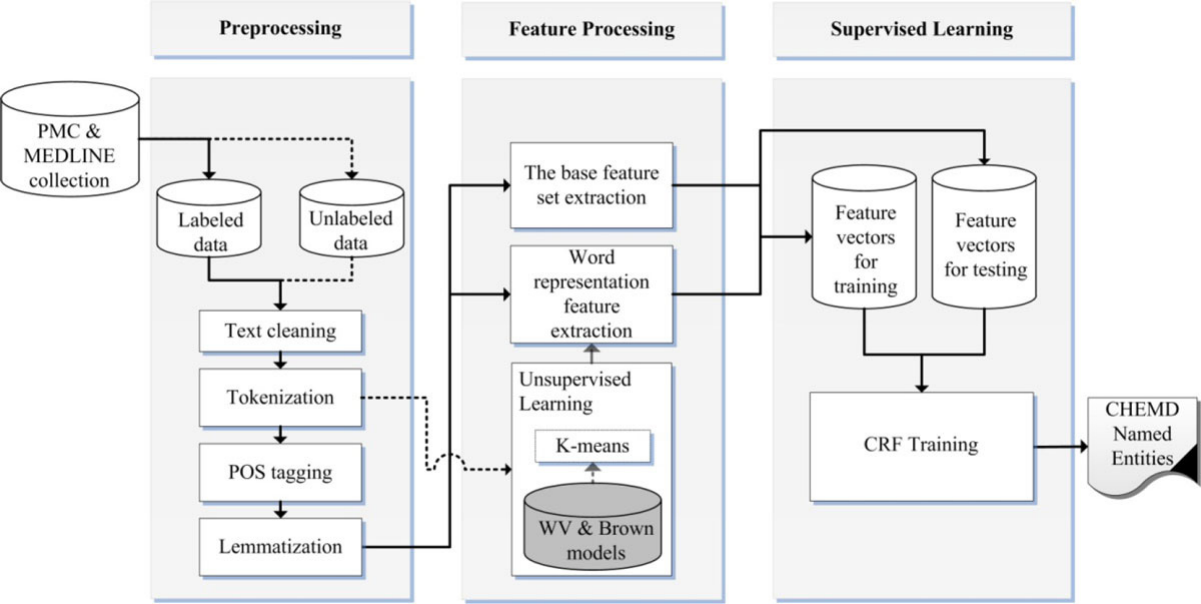
**System design for chemical and drug Named Entity Recognition**. The solid lines represent the flow of labeled data, and the dotted lines represent the flow of unlabeled data.

Our chemical and drug NER system design is shown in Figure 1. First, we perform preprocessing on MEDLINE and PMC document collection and then extract two different feature sets, a base feature set and a word representation feature set, in the feature processing phase. The unlabeled set of the collection is fed to unsupervised learning of the feature processing phase to build word classes. Finally, we apply the CRF sequence-labeling method to the extracted feature vectors to train the NER model. These steps will be described in subsequent sections.

### Preprocessing

Preprocessing is where text data is cleaned and processed via NLP tasks and is a preparatory task for feature processing.

First, the text data is cleansed by removing non-informative characters and replacing special characters with corresponding spellings. The text is then tokenized with a tokenization tool. We evaluated two different tokenization strategies: a simple white space tokenizer and the BANNER simple tokenizer. The white space tokenizer splits the text simply, based on blanks within it, whereas the BANNER tokenizer breaks tokens into either a contiguous block of letters and/or digits or a single punctuation mark. Finally, the lemma and the part-of-speech (POS) information were obtained for a further usage in the feature extraction phase. In BANNER-CHEMDNER, BioLemmatizer [27] was used for lemma extraction, which resulted in a significant improvement in overall system performance.

In addition to these preprocessing steps, special care is taken to parse the PMC XML documents to get the full text for the unlabeled data collection.

### Feature processing

We extract features from the preprocessed text to represent each token as a feature vector, and then an ML algorithm is employed to build a model for NER.

The proposed method includes extraction of the baseline and the word representation feature sets. The baseline feature set is essential in NER, but is poor at representing the domain background because it only carries some morphological and shallow-syntax information of words. On the other hand, the word representation features can be extracted by learning on a large amount of text and may be capable of introducing domain background to the NER model.

The entire feature set for a token is expanded to include features for the surroundings with a two-length sliding window. The word, the word *n*-gram, the character *n*-gram, lemma and the traditional orthographic information are extracted as the baseline feature set. The regular expressions that reveal orthographic information are matched to the tokens to give orthographic information. These baseline features are summarized in Table 1.

**Table 1:** The baseline features.

Feature description	Note/Regular expression
Roman number	[ivxdlcm]+|[IVXDLCM]+

Punctuation	[,\\.;:?!]

Start with dash	"-.*

Nucleotide sequence	[atgcu]+

Number	[0-9]+

Capitalized	[A-Z] [a-z]*

Quote	[\"`']

The lemma for the current token	Provided by BioLemmatizer [23]

2, 3 and 4-character prefixes and suffixes	

2 and 3 character n-grams	Token start or end indicators are included

2 and 3 word n-grams	

For word representation features, we train Brown clustering models [28] and Word Vector (WV) models [17] on a large PubMed and PMC document collection. Brown clustering is a hierarchical word clustering method, grouping words in an input corpus to maximize the mutual information of bigrams. Therefore, the quality of a partition can be computed as a sum of mutual information weights between clusters. It runs in time O(V × K^2^), where V is the size of the vocabulary and K is the number of clusters.

The VW model is induced via a Recurrent Neural Network (RNN) and can be seen as a language model that consists of *n*-dimensional continuous valued vectors, each of which represents a word in the training corpus. The RNN instance is trained to predict either the middle word of a token sequence captured in a window (CBOW) or surrounding words given the middle word of the sequence (skip-gram) depending on the model architecture [17]. The RNN becomes a log-linear classifier, once its non-linear hidden layer is removed, so the training process speeds up allowing millions of documents to process within an hour. We used a tool implemented by Mikolov et al. [17] to build our WV model from the PubMed collection.

Further, the word vectors are clustered using a K-means algorithm to drive a Word Vector Class (WVC) model. Since Brown clustering is a bigram model, this model may not be able to carry wide context information of a word, whereas the WVC model is an *n*-gram model (usually *n *= 5) and learns broad context information from the domain corpus. We drive the cluster label prefixes with 4, 6, 10 and 20 lengths in the Brown model by following the experiment of Turian et al. [16], and the WVC models induced from 250-dimension WVs as word representation features.

For feature extraction, we do not rely on any lexicon nor any dictionary other than the free text in the domain in order to keep the system applicable to other NER tasks in bio-text data, even though the usage of such resources is reported to considerably boost system performance. Most of the top performing systems participated in CHEMDNER task use the domain lexicon and observed a considerable performance boost [29].

### Supervised learning

CRF - a probabilistic undirected graphical model has been used successfully in a large number of studies on NER, because it takes advantage of sequence labelling by treating each sentence as a sequence of tokens. We apply a second-order CRF model, where the current label is conditioned on the previous two using a Begin, Inside, Outside (BIO) tagging format of the tokens. In the BIO tagging format, each token is classified either at the beginning, inside or outside of a named entity, and a postprocessing task forms the named entity mentions by merging the tagged tokens.

We use a Machine Learning for Language Toolkit (MALLET) library [30] for training the CRF model, because the BANNER system provides a convenient interface to work with it. The BANNER system also includes two types of general postprocessing that could be useful for any NER tasks in bio-text data. The first type is based on the symmetry of parenthesis, brackets or double quotation marks. Since these punctuation marks are always paired, BANNER drops any named entity mention containing mismatched parentheses, brackets or double quotation marks. The second type of postprocessing is dedicated to resolving abbreviations of named entities.

## Results

### Dataset

Because the proposed method is a semi-supervised learning method exploiting unlabeled data during feature extraction, we prepared a large document collection of domain text in addition to annotated datasets.

We evaluated the system for chemical and drug NER with a CHEMDNER dataset provided by the BioCreative IV CHEMDNER task organizers. The dataset consists of 10,000 annotated documents subdivided into training and development sets of 3,500 documents each, and a testing set of 3,000 documents. The BioCreative II Gene Mention (BC2GM) dataset was used to compare the system against existing systems of the biomedical NER. The BC2GM dataset originally splits into subsets of 15,000 and 5,000 sentences for training and testing, respectively.

For the unlabeled data, we collected around 1.4 million PubMed abstracts and full text articles from the whole PMC database available at the time (over 2 million documents). After preprocessing, we derived two different text corpora: a PubMed abstract corpus consisting of a vocabulary of 1,136,085 entries for induction of Brown clustering models, and a merged corpus of both resources with a vocabulary of 4,359,932 entries for training WV models. Given the limited resources and time, we were able to induce the Brown clustering models only with the PubMed abstract corpus. We would like to build a Brown model with the full corpus. However, this would take several months, making it impossible to test on the CHEMDNER testing set in the given period.

### Evaluation measure

For both CDI and CEM subtasks, an exact matching criteria was used to examine three different result types. False negative (*FN*) and False positives (*FP*) are incorrect negative and positive predictions. True positives (*TP*) results corresponded to correct positive predictions, which are actual correct predictions. The evaluation is based on the performance measures *p *(precision), *r *(recall), and *F*.

Recall denotes the percentage of correctly labeled positive results over all positive cases and is calculated as: *r *= *TP/(TP+FN)*.

Precision is the percentage of correctly labeled positive results over all positive labeled results and is calculated as: *p *= *TP/(TP+FP)*.

The F-measure is the harmonic average of precision and recall, and a balanced F-measure is expressed as: *F_1 _= 2pr/(p+r)*.

### Chemical and drug named entity recognition

We trained the second-order CRF models with different features on the training set and evaluated the models on the development set. Consequently, after noticing the best settings for the hyperparameters, we trained the models on a merged set of the training and the development sets and reported the performance on the testing set. Table 2 and Table 3 show the performance comparison of the different runs with varied feature settings for CDI and CEM subtasks. Both tasks are interconnected, and their performance measures are interchangeable.

**Table 2:** CDI subtask evaluation results of different runs with varied features.

	Development set			Testing set		
	
Features	Pre	Rec	F-scr	Pre	Rec	F-scr
BANNER setup	82.83	78.71	80.72	85.36	85.29	85.32

Baseline	81.71	82.3	82	75.87	70.55	73.11

Baseline + Brown 300	82.2	82.96	82.58	86.03	85.45	85.74

Baseline + Brown 1000	81.96	83.24	82.59	86.04	85.60	85.82

Baseline + Brown 1000 + WVC 1000	82.73	83.89	83.31	86.23	85.37	85.8

Baseline + Brown 1000 + Brown 300	82.1	83.42	82.76	86.46	85.63	86.04

Baseline + Brown 1000 + WVC 300	82.43	83.82	83.12	86.06	86.06	86.06

Baseline + Brown 1000 + WVC 500	82.78	83.56	83.17	86.12	86.2	86.16

Baseline + Brown 1000 + WVC 500 + WVC 300	**83.1**	83.78	**83.44**	86.10	86.31	86.2

Baseline + Brown 1000 + WVC 500 + WVC 1000	82.78	83.76	83.27	86.19	86.4	86.28

Baseline + Brown 1000 + WVC 500 + WVC 300 + WVC 1000	82.3	**84.05**	83.16	**86.47**	**86.47**	**86.47**

Feature groups are separated by (+). The parameters followed Brown and WVC are the number of classes induced in each model. Pre: Precision, Rec: Recall, F-scr: F-score.

**Table 3:** CEM subtask evaluation results of different runs with varied features.

	Development set			Testing set		
	
Features	Pre	Rec	F-scr	Pre	Rec	F-scr
BANNER setup	85.59	72.74	78.64	88.2	80.74	84.31

Baseline	84.40	77.34	80.71	79.81	63.16	70.51

Baseline + Brown 300	84.6	78.47	81.42	88.67	81.17	84.75

Baseline + Brown 1000	84.6	79.34	81.89	88.71	81.39	84.89

Baseline + Brown 1000 + WVC 1000	85.25	80.3	82.7	88.79	81.45	84.96

Baseline + Brown 1000 + Brown 300	84.76	79.46	82.03	89.1	81.54	85.2

Baseline + Brown 1000 + WVC 300	84.98	80.07	82.45	88.65	82.13	85.26

Baseline + Brown 1000 + WVC 500	85.32	79.92	82.53	88.77	82.42	85.48

Baseline + Brown 1000 + WVC 500 + WVC 300	**85.58**	80.1	**82.75**	88.57	82.6	85.48

Baseline + Brown 1000 + WVC 500 + WVC 1000	85.28	80.28	82.7	88.8	82.6	85.59

Baseline + Brown 1000 + WVC 500 + WVC 300 + WVC 1000	84.89	**80.35**	82.56	**88.9**	**82.68**	**85.68**

Feature groups are separated by (+). The parameters followed Brown and WVC are the number of classes induced in each model. Pre: Precision, Rec: Recall, F-scr: F-score.

We started conducting a run with a basic feature setting and a BANNER setup, and gradually increased the complexity of the feature space for further runs. The inclusion of lemma by BioLemmatizer as a feature (baseline feature setup) in addition to BANNER feature set yielded a significant improvement on the development set. Surprisingly, the model based on the baseline features converged to an optimum, possibly a local optimum in the first 30 iterations of the training, and reported a worthless performance when evaluating the testing set. A Brown model with a larger number of clusters tended to obtain a higher F-measure. Unlike Brown clustering, a large or a lower number of WVCs degraded the performance. We found the WVC model with 500 different classes the best performing one on this task. During the induction of the WVC models, we set the WV dimension to 250 which is a trade-off value between computational cost and WV quality. Further, the combination of the different WVC models significantly improved the F-measure. We achieved the best performance, an 85.68% F-measure for CEM and an 86.47% F-measure for CDI subtasks, with the model based on the baseline feature set, the 1000-Brown clustering, and 300, 500 and 1000 WVCs (the baseline + Brown 1000 + WVC 300 + WVC 500 + WVC 1000 setup). This result is 0.93% and 0.73% higher than our best entries for CHEMDNER CEM and CDI subtasks [31]. The word representation features extracted from the unlabeled data boosted performance by a 1.37% and a 1.14% F-measure for CEM and CDI subtasks, respectively.

### Gene and protein mention recognition

We trained BANNER-CHEMDNER on the BC2GM training set and evaluated it on the testing set, following the convention of the BioCreative II gene mention task. Table 4 lists the different runs with varied features included in the NER model. We expected similar results to be obtained in chemical and drug NER. However, the combination of the features did not show the expected improvement, and the best result (an 87.04% F-measure) that we obtained was a run with a model based on the baseline feature set, the 1000-Brown clustering and 500 WVCs (baseline + Brown 1000 + WVC 500).

**Table 4:** BioCreative II gene mention evaluation results of different runs with varied features.

	Testing set		
	
Features	Pre	Rec	F-scr
Baseline + Brown 300	86.49	83.79	85.12

Baseline	86.88	84.09	85.47

Baseline + Brown 1000	86.82	84.27	85.53

Baseline + Brown 1000 + WVC 500 + WVC 300	87.09	84.98	86.02

Baseline + Brown 1000 + WVC 300	87.95	84.27	86.07

Baseline + Brown 1000 + WVC 500 + WVC 1000	87.92	85.49	86.69

Baseline + Brown 1000 + WVC 1000	**88.13**	85.58	86.84

Baseline + Brown 1000 + WVC 500	88.02	**86.09**	**87.04**

Feature groups are separated by (+). The parameters followed Brown and WVC are the number of classes induced in each model. Pre: Precision, Rec: Recall, F-scr: F-score.

We finally compared our best result to the task entries and the BANNER system result (Table 5).

**Table 5:** Comparison of different systems on the BioCreative II testing set.

System or author	BioCreative II rank	Pre	Rec	F-scr
Ando[32]	1	88.48	85.97	87.21

BANNER-CHEMDNER	-	88.02	86.08	87.04

Kuo et al. [33]	2	89.3	84.49	86.83

Huang et al. [34]	3	84.93	88.28	86.57

BANNER	-	88.66	84.32	86.43

Ando focused on a semi-supervised approach, alternating structure optimization. The system learns better feature representations from a large collection of PubMed text and uses a regularized linear classifier during the supervised training on annotated data. Kuo et al. [33] utilized domain lexicons and bi-direction parsing models of CRFs. The results from left-right and right-left parsing models were combined to produce a set of higher recall mention answers. Huang et al. [34] explored an ensemble of SVM and CRFs models. They applied intersection to the tagging results of the two SVM models and then union with the tagging results of the CRF model in their ensemble approach.

## Discussion

Several different runs other than the ones reported in the results section were carried out. We observed that the whitespace tokenizer performs better than the BANNER simple tokenizer for extraction of word representation features. A model with an additional feature set of raw WVs was trained and evaluated. However, we found that the word WVs did not always improve performance, and in some cases, degraded system performance with the CRF model. That is, the continuous valued WV features add some level of complexity to the model, overfitting the CRF model. Even though the WV features with the CRF model did not achieve improvement, those continuous valued features could be useful in conjunction with other classifiers, such as perceptron and support vector machines [8]. Another finding is that the Brown cluster features always improve F-measure, and the improvement is significant when the model is built on the domain text corpus.

## Conclusions

We proposed a semi-supervised learning method that exploits unlabeled data efficiently in order to incorporate domain knowledge into a Named Entity Recognition model and to leverage overall system performance. The key feature of the method is learning word representations from a large amount of text data for feature extraction. The generally applicable word representation features were reported to boost system performance significantly for both chemical and biomedical NER.

We extended BANNER, a biomedical NER system, with the proposed method. This yields an integrated system that can be applied for chemical and drug NER or biomedical NER. We call our branch of the BANNER system BANNER-CHEMDNER.

BANNER-CHEMDNER achieves an 85.68% and an 86.47% F-measure on the testing set for CHEMDNER CEM and CDI subtasks, respectively, and an 87.04% F-measure on the official testing set of the BioCreative II gene mention task, showing remarkable performance for both chemical and biomedical NER.

Our future work should be towards a unified NER system in biomedical, chemical and medical domain, based on the generally applicable word representations.

## Competing interests

The authors declare that they have no competing interests.

## Authors' contributions

TM conceived the study, participated in its design, developed the extension program, and drafted the manuscript. ML carried out calculations and helped draft the manuscript. KB participated in data analysis and helped draft the manuscript. HAP participated in study design. NHC helped draft the manuscript. KHR provided system design, valuable guidance, editing, and research grant. All authors read and approved the final manuscript.
